# Estimation of the tissue and serum levels of IL-35 in Mycosis fungoides: a case-control study

**DOI:** 10.1007/s00403-024-03115-9

**Published:** 2024-06-08

**Authors:** Maha Fathy Elmasry, Yasmine Ahmed Obaid, Solwan Ibrahim El-Samanoudy, Zeinab Ahmed Nour, Sally Sameh Doss

**Affiliations:** 1 Dermatology Department, Faculty of Medicine, Cairo University, Cairo, Egypt; 2 Medical Biochemistry and Molecular Biology Department, Faculty of Medicine, Cairo University, Cairo, Egypt

**Keywords:** Mycosis fungoides, Interleukin (IL-35), Cutaneous T- cell lymphoma (CTCL), Pathogenesis, Lymphocytes

## Abstract

Mycosis fungoides (MF) is the most common primary cutaneous T-cell lymphoma (CTCL) with its etiology not yet fully understood. Interleukin (IL)-35 is an inhibitory cytokine that belongs to the IL-12 family. Elevated IL-35 in the plasma and the tumor microenvironment increases tumorigenesis and indicates poor prognosis in different types of malignancies. The objective of this study is to estimate the expression levels of IL-35 in tissue and serum of MF patients versus healthy controls. This case-control study included 35 patients with patch, plaque, and tumor MF as well as 30 healthy controls. Patients were fully assessed, and serum samples and lesional skin biopsies were taken prior to starting treatment. The IL-35 levels were measured in both serum and tissue biopsies by ELISA technique. Both tissue and serum IL-35 levels were significantly higher in MF patients than in controls (*P* < 0.001) and tissue IL-35 was significantly higher than serum IL-35 in MF patients (*P* < 0.001). Tissue IL-35 was significantly higher in female patients and patients with recurrent MF compared to male patients and those without recurrent disease (*P* < 0.001). Since both tissue and serum IL-35 levels are increased in MF, IL-35 is suggested to have a possible role in MF pathogenesis. IL-35 can be a useful diagnostic marker for MF. Tissue IL-35 can also be an indicator of disease recurrence.

**Supplementary Information:**

The online version contains supplementary material available at 10.1007/s00403-024-03115-9.

## Introduction

The most frequent form of primary cutaneous lymphoma is mycosis fungoides (MF). lt is postulated to derive from mature skin-resident effector memory T cells [1]. The majority of patients with MF are patch and plaque phases, and a minority advance to the tumor stage. MF often has a protracted and indolent course [2].

The etiopathogenesis of CTCL remains largely unknown. Genomic analysis of CTCL cell lines has shown genetic, epigenetic, and chromosomal aberrations that can affect lymphomagenesis and consequently, disease progression. The uncontrolled clonal proliferation of atypical lymphocytes results from chronic antigenic stimulation, together with the underlying cytogenetic abnormalities [3]. Neoplastic T-cell clones in CTCL secrete Th2-associated cytokines that lead to progressive immune dysregulation and tumor growth [4].

Interleukin-35 (IL-35) is an inhibitory cytokine belonging to the IL-12 family. It can suppress T cell proliferation and induce IL-35-producing induced-regulatory T cells (iTr35) which inhibits inflammatory responses [5].

IL-35 was thought to be produced by activated Tregs but more recent studies revealed the expression of IL-35 by other immunoregulatory cells as regulatory B cells (Bregs), tolerogenic DCs (tolDCs), prometastatic tumor-associated macrophages, and CD8 + T regulatory cells [5].

The effect of IL-35 on the neoplastic cells and the relationship between its expression rate and different stages of cancer is currently unknown [6]. High levels of IL‐35 in the plasma tumor microenvironment increase tumorigenesis and denote a poor prognosis in many neoplasms [7].

IL-35 increases tumorigenesis by inhibiting the antitumor activity of lymphocytes which leads to the accumulation of CD11b + Gr1 + myeloid cells (MDSC), also tumor-produced IL‐35 has an indirect role in MDSC chemotaxis. The elevated MDSC produces vascular endothelial growth factor (VEGF) together with other factors that promote tumor angiogenesis and tumor cell expansion [6]. In addition, IL-35 in tumor-infiltrating Treg cells can promote the expression of programmed cell death protein-1 (PD-1) on the surface of the tumor T cells, leading to their exhaustion [8].

## Subjects and methods

This study was designed as a case-control study. It was conducted at the Dermatology outpatient clinic, Dermatology department, Faculty of Medicine, Cairo University Hospital (Kasr Al Ainy) from December 2022 to June 2023. The Dermatology Research Ethical Committee had approved this study’s protocol. Confidentiality in handling the data was guaranteed. A written informed consent was signed by each patient participating in the study. Clinical trial ID: NCT05855460.

### Patients and controls

Thirty-five MF patients (≥ 18 years old) including both sexes, were recruited, as well as 30 age- and sex-matched healthy controls.

The diagnosis of MF was confirmed histopathologically by two skin biopsies and patients were properly clinically assessed and investigated by chest X-ray, abdominal, and lymph node ultrasonography for staging their MF by the TNM staging. All patients with classic MF including patch, plaque, and tumor stages were included. Patients with any other skin disease, patients receiving topical treatment, systemic treatment, or phototherapy for MF during the past 3 months and patients with a history of a solid or hematological malignancy were excluded from this study.

## Methods

For assessment of tissue levels of IL35, a 4 mm punch skin biopsy was taken from every patient’s lesional skin and healthy control and kept incubated in phosphate-buffered saline (PBS) at -80 degrees till assayed. For assessment of serum levels of IL35, 5 ml venous blood was taken from every patient and healthy control and centrifuged at 8000 RPM for 20 min, and then serum was separated and kept frozen at -80 degrees till assayed. The serum and tissue levels of Human Interleukin-35 were assayed by a commercially available ELISA kit supplied by SUNLONG BIOTECH Co., Ltd., China. Catalog No. SL1009Hu. Details of laboratory methods and statistical analysis are illustrated in the supplementary file.

## Results

This study included 35 MF patients and 30 healthy controls. Both groups were age- and sex-matched (*P* > 0.05). MF patients included 15 male (42.9%) and 20 female (57.1%) patients, and their ages ranged between 22 and 73 years with a mean of 48.69 ± 14.79 years. Meanwhile, healthy controls included 16 females (53.3%) and 14 males (46.7%) and their ages ranged between 20 and 68 years with a mean of 42.47 ± 14.81 years. The clinical data of patients are shown in Table 1, and the laboratory investigations of patients are illustrated in **Suppl. Table 1.**

**Table 1:** Clinical data of the patients

		No.	%
Recurrence	Yes	16	45.7%
	No	19	54.3%
Type of lesion	Tumor	5	14.3%
	Plaque	9	25.7%
	Patch	21	60.0%
Stage	IA	6	17.1%
	IB	14	40.0%
	IIA	10	28.6%
	IIB	4	11.4%
	IIIA	1	2.9%
	**Mean ± SD**	**Median**	**Range**
Duration (months)	55.3 **±** 71.6	36	(1-360)
BSA (%)	49.5 **±** 28.9	50	(5–95)

BSA: body surface area, SD: standard deviation.

**Fig. 1:**
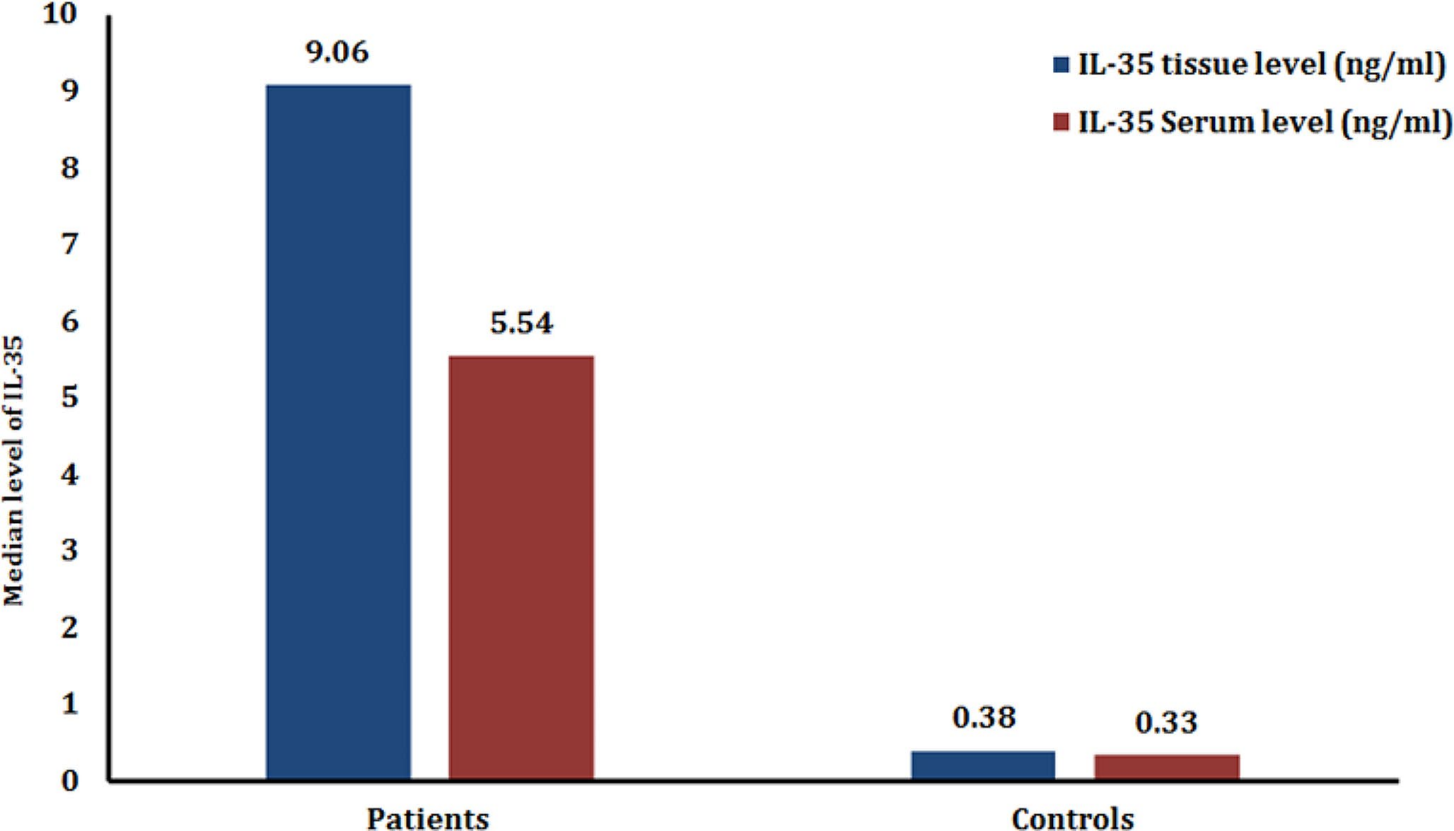
Bar chart of IL-35 tissue and serum levels among patients versus controls

The levels of both tissue and serum levels of IL-35 were significantly higher in MF patients than in healthy controls (*P* < 0.001) **(**Table 2**) (**Fig. 1**).**

**Table 2:** Comparison between tissue and serum levels of IL-35 in the two studied groups:

	Patients (*n* = 35)	Controls (*n* = 30)	*P* value
IL-35 tissue level (ng/ml) (Mean ± SD)	8.96 ± 1.25	0.39 ± 0.07	< 0.001*
IL-35 serum level (ng/ml) (Mean ± SD	7.36 ± 8.07	0.34 ± 0.07	< 0.001*

SD: standard deviation, *P < 0.001 is highly significant, Analysis done by Mann Whiteny test.

A receiver operator characteristic (ROC) curve was done to detect the cutoff value, sensitivity, and specificity of tissue and serum IL-35. Both were found as a significant diagnostic marker of MF (*P* < 0.001) with tissue IL-35 area under the curve (AUC) was 1.000 (cutoff value 2.5318 ng/ml, 95% confidence interval 1.000–1.000 with sensitivity 100% and specificity 100%) and serum IL-35 AUC was 1.000 (cutoff value 1.0036 ng/ml, 95% confidence interval 1.000–1.000 with sensitivity 100% and specificity 100%). The positive predictive value (PPV) was 100%, The negative predictive value (NPV) was 100% and the accuracy was 100%. The results demonstrate that both IL-35 tissue, and serum levels have excellent diagnostic accuracy, as indicated by the AUC of 1.00. The high sensitivity, specificity, PPV, NPV, and accuracy values further support the reliability of IL- 35 levels as a diagnostic marker **(Suppl. Table 2)**.

**Fig. 2:**
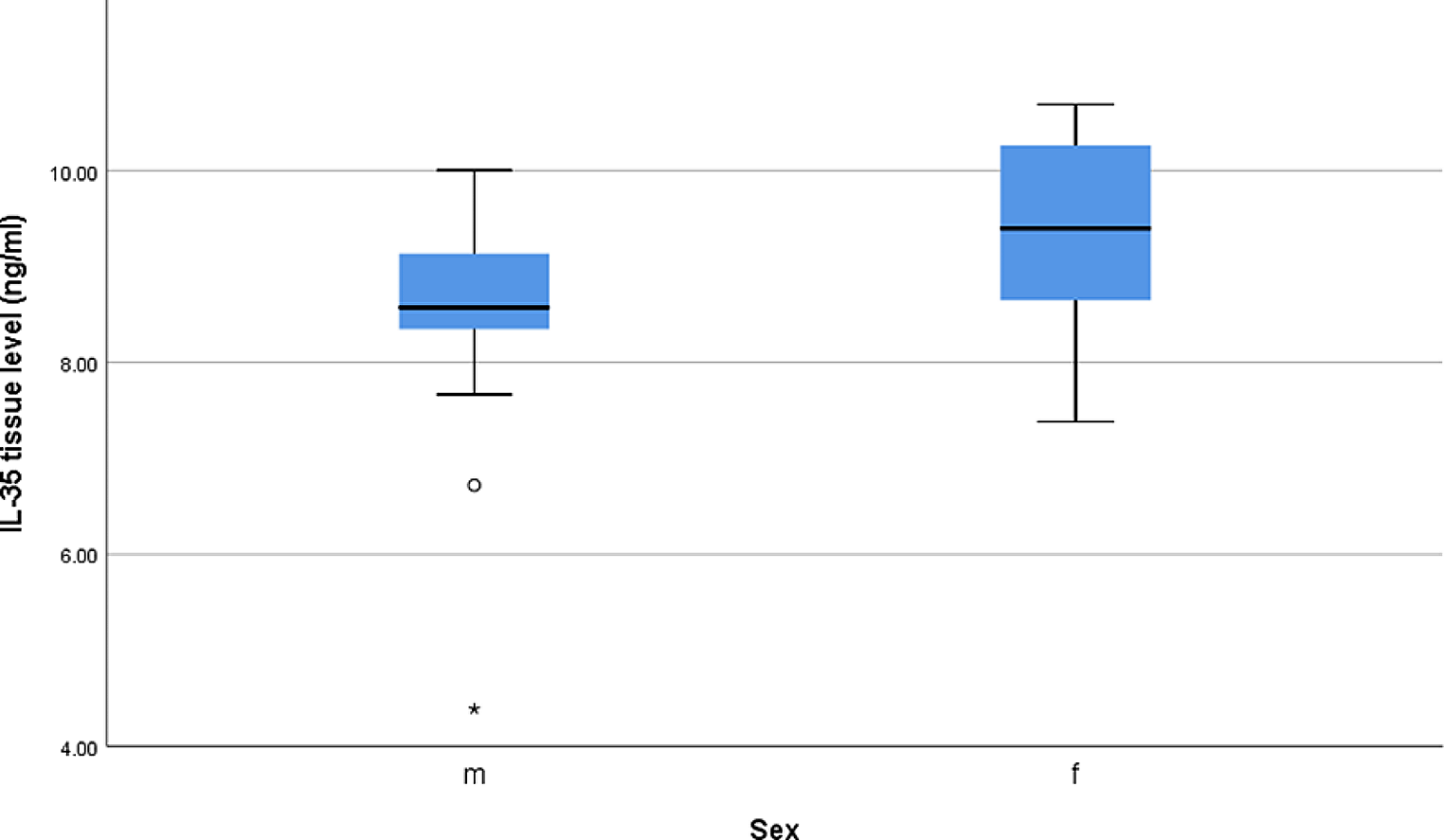
Box plot of IL-35 tissue levels among males and females in the studied patients

**Fig. 3:**
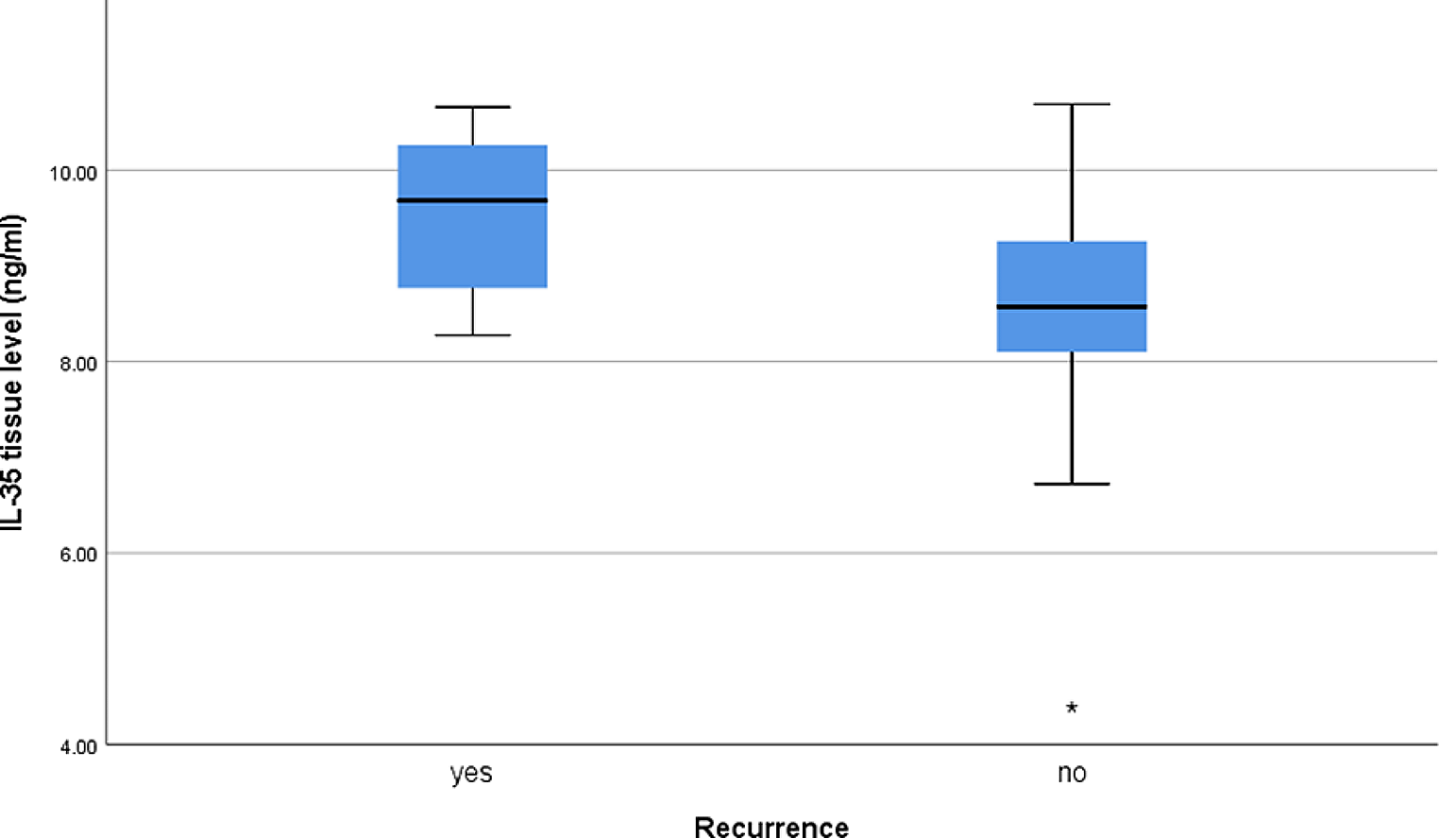
Box plot of IL-35 tissue levels among patients with recurrent MF and without recurrent disease

Regarding IL-35 tissue level in the patients’ group, a statistically significant difference was found between males and females *(P* < 0.001*)*, with higher levels in females **(**Fig. 2**).** Also, the difference in the level between patients with and without recurrent disease was statistically significant (*P* < 0.001***)*** with a higher level in patients with recurrent disease **(**Fig. 3**).** However, no statistically significant differences were found in IL-35 tissue level among different stages, type of lesions, or different skin types (*P* > 0.05) **(Suppl. Table 3).**

Regarding IL-35 serum levels in the patients’ group, no statistically significant differences were found in IL-35 serum level and sex, skin types, among different stages, types of lesions, or recurrent disease (*P*˃0.05) **(Suppl. Table 4).**

No correlations were found between either IL-35 tissue or serum levels and age (of patients or controls), duration of disease, or BSA % (*P*˃0.05). Also, no statistically significant difference was found in either IL-35 tissue or serum levels between males and females in the control group (*P* > 0.05) **(Suppl. Tables 5,6).**

Regarding patients’ laboratory investigations, IL-35 tissue level showed only a statistically significant negative correlation with monocyte count (*P* = 0.014, *r*=-0.412). Also, there was a statistically significant positive correlation between IL- 35 serum level and platelet count (*P* = 0.008, *r* = 0.442). No correlations were found between either tissue or serum IL-35 and any other laboratory investigation (*P*˃0.05) **(Suppl. Tables 5,6).**

**Fig. 4:**
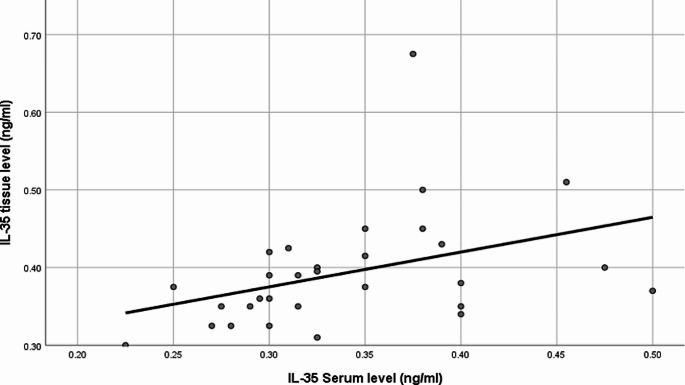
Scatter plot of correlation between IL-35 tissue levels and IL-35 serum levels

A statistically significant positive correlation was found between tissue and serum IL-35 levels in controls (*P* = 0.006, *r* = 0.491) **(**Fig. 4**).** This positive correlation was not significant among the MF group.

## Discussion

The mechanisms involved in the progression of MF remain unclear. Different factors could be involved such as aberrant molecular expression, overexpression of microRNA, changes in release of cytokines, genetic mutations, and different compositions of cells in the tumor microenvironment [9].

Tumor microenvironment changes are due to the production of different cytokines from the tumor cells. This eventually leads to a shift in the immune response from an anti-tumor (Th1) to a tumorigenic (Th2) one [10]. This shift will increase the immunosuppressive cytokines (IL-2, IL-4, IL-7, IL-13, and IL-15) production by neoplastic elements which lead to tumor growth and spread [11].

Interleukin-35 (IL-35) is an IL-12 family cytokines member, but unlike other IL-12 family members, it has immuno-suppressive activity [12].

The immune-suppressive cytokines lead to alteration in the epigenome and the transcriptional network in naïve immune cells [13]. These cytokines act on cytotoxic CD8 + T cells or natural killer (NK) cells and inhibit their effector functions and proliferation into the tumor microenvironment [14]. The immunosuppressive cytokines, TGF-β, IL-4, IL-10, and IL-35, are the cytokines that are dominant in the tumor environment in different tumors. Targeting these cytokines can limit the survival of tumor cells and control tumor growth and metastasis [15].

The role of IL-35 in MF has not been studied before; accordingly, this study was designed to assess the IL-35 level in lesional skin and serum of 35 MF patients versus 30 healthy controls and statistically significant differences between the studied groups were detected regarding the levels of tissue and serum IL-35. These levels were found to be significantly higher in MF patients than in the healthy controls. These findings were augmented by unadjusted and adjusted regression analysis which proved that both serum and tissue IL-35 are significant diagnostic markers for MF.

Our results are in concordance with the findings of previous studies that had detected elevated levels of IL-35 in different tumors such as diffuse large B-cell lymphoma [16], acute myeloid leukemia [17], acute lymphoblastic leukemia [18], colorectal carcinoma [19], pancreatic adenocarcinoma [20], gastric carcinoma [21, 22], prostate carcinoma [23], breast carcinoma [24], non-small cell lung cancer [25], and hepatocellular carcinoma [26].

Interleukin-35 also inhibits immune responses by promoting regulatory T cells (Tregs) and regulatory B cells (Bregs) expansion and at the same time suppressing macrophages, effector T cells, Th1 cells, and Th17 cells [27]. Also, IL-35 has been found to affect the secretion of other cytokines such as IL-6, granulocyte colony-stimulating factor (G-CSF), and IFN-γ eventually leading to a protumor effect [27].

Although higher levels of IL-35 were linked to disease progression and metastasis in other studies, our study didn’t find statistically significant differences between different stages of MF. This may be due to the low number of patients in advanced stages included in the study. So, further studies are needed with a larger number of patients in advanced stages to assess its prognostic value, especially patients with blood involvement.

Moreover, a study revealed that recombinant IL-35 stimulates PD-1 in peripheral CD8 + T cells as well as those infiltrating the tumour in patients with hepatocellular carcinoma (HCC). This shows that IL-35 and its antibody could be potential therapeutic agents in different neoplasms and immune-mediated diseases [28].

In the current study, tissue levels of IL-35 were significantly higher in females. This is mostly related to high estrogen levels. Polanczyk et al. [29] reported that estrogen has immunomodulatory and immunosuppressive functions by reducing activation of effector T cells, potentiation of Treg cells, inhibiting the induction of the inflammatory cytokines as IL-12 and interferon-gamma but enhancing the secretion of the immunosuppressive cytokines as IL-10 and enhancing the expression of the PD-1 costimulatory pathway.

Moreover, the level of tissue IL-35 was significantly higher in patients with recurrent MF. The higher level of IL-35 in relapsing disease may be related to the clonal expansion of malignant T cells and the dysregulated immune response in relapsing cancers. In MF, the malignant T cells evade immune surveillance through different mechanisms such as downregulation of major histocompatibility complex molecules, impaired antigen presentation, and the production of immunosuppressive cytokines. These immune evasion strategies allow the malignant T cells to escape recognition and elimination by the immune system, contributing to disease relapse [30]. Therefore, tissue IL-35 may be of value as a potential marker to pick up very early recurring cases.

Regarding patients’ laboratory investigations, IL-35 tissue level showed a statistically significant negative correlation with monocyte count. In CTCL, macrophage-related chemokines and angiogenic factors produced by tumor-associated macrophages (TAMs), developing from tissue-infiltrating monocytes, have crucial roles in tumor formation in the lesional skin of MF. The TAMs maintain an immunosuppressive tumor microenvironment by recruiting Tregs, circulating myeloid cells, and myeloid-derived suppressor cells [31]. In the same context, both subunits of IL-35 (EBI3 and IL-12a) are highly expressed in metastatic TAMs [32].

Also, there was a statistically significant positive correlation between IL- 35 serum level and platelet count. This could be attributed to the active role of platelets (rich in angiogenic factors in their granules) in the tumor microenvironment (TME), being involved in tumor angiogenesis as they are rich in angiogenic factors in their granules [33]. Similarly, IL-35 promotes tumor angiogenesis and tumor cell expansion through inducing the formation of VEGF [6].

No correlations were found between either tissue or serum IL-35 and any other laboratory investigation (*P*˃0.05) although some studies reported significant correlations suggesting MF association with metabolic syndrome and cardiovascular risk [34, 35].

Also, a statistically significant difference was detected between the level of tissue and serum IL-35 in patients, with higher levels in tissue. This may point out that tissue IL-35 level is more accurate as a diagnostic marker in MF than the serum level.

## Conclusion

In conclusion, being significantly higher in MF patients compared to healthy controls, IL-35 is suggested to play a possible role in MF pathogenesis. IL-35 can be a useful diagnostic marker in MF. Our study found that tissue IL-35 was significantly higher in patients with recurrent MF compared to de novo disease. So, tissue IL-35 can also be an early indicator for disease recurrence in suspicious lesions.

In line with the promising use of anti-IL-35 antibodies in different cancers, anti-IL-35 antibodies may have a promising role as a potential therapeutic agent for MF.

## Electronic supplementary material

Below is the link to the electronic supplementary material.

Supplementary Material 1

## Data Availability

The data supporting findings of this study are available within the article. Raw data from which the findings of this study were obtained, are available from the corresponding author, upon request.
