# Supplementary material for: Estimation of the tissue and serum levels of IL-35 in Mycosis fungoides: a case-control study
Source: Arch Dermatol Res. 2024 Jun 8;316(7):349. doi: 10.1007/s00403-024-03115-9 (PMC11162372; doi:10.1007/s00403-024-03115-9)
Supplement: Supplementary file 1 — Supplementary Material 1 [file 403_2024_3115_MOESM1_ESM.docx]

**Statistical analysis:**

Data were coded and entered using the statistical package for the Social Sciences (SPSS) version 28 (IBM Corp., Armonk, NY, USA). Data was summarized using mean, standard deviation, median, minimum and maximum in quantitative data and using frequency (count) and relative frequency (percentage) for categorical data. Comparisons between quantitative variables were done using the non-parametric Kruskal-Wallis and Mann-Whitney tests. For comparison of paired measurements within each patient the non-parametric Wilcoxon signed rank test was used. For comparing categorical data, Chi square (χ2) test was performed. Exact test was used instead when the expected frequency is less than 5. Correlations between quantitative variables were done using Spearman correlation coefficient. ROC curve was constructed with area under curve analysis performed to detect best cutoff value of IL 35 for detection of cases. P-values less than 0.05 were considered statistically significant.

**Laboratory work:**

**Skin biopsy:**

The skin biopsies were stored at -20^o^C in eppendorfs with 300 ul phosphate buffer saline (PBS) added to each eppendorf. Tissue samples with PBS were homogenized by a grinder, then centrifuged for 20 minutes at 3000 revolutions per minute (rpm) speed, the supernatant was removed to be added to the ELISA kit wells for Human Interleukin-35 assay.

**Estimation of Serum and Tissue Human Interleukin-35**

The serum and tissue levels of Human Interleukin-35 were assayed by commercially available ELISA kit supplied by SUNLONG BIOTECH Co., Ltd., China. Catalog No. SL1009Hu.

**Principle of the procedure:**

The test principle is Sandwich enzyme immunoassay. The microtiter plate has been pre-coated with an antibody specific to human IL-35. Standards and samples are then added to the appropriate microtiter plate wells combined to the specific antibody then a Horseradish Peroxidase (HRP) IL-35 conjugated antibody specific for IL-35 is added to each microplate well and incubated. Free components are washed away. The TMB substrate solution is added to each well, only those wells that contain human IL-35 and HRP-conjugated IL-35 antibody will appear blue in color and then turn yellow after adding the stop solution to stop the coloring reaction. The color change is measured spectrophotometrically at a wavelength of 450nm ± 2nm.

**Procedure steps:**

- 1. Standards, samples and controls were added to the wells.
  2. The plate was left to incubate for 30 minutes at 37°C.
  3. After incubation, a washing step was done to remove unbound and nonspecifically bound molecules.
  4. HRP-conjugate reagent was added to each well**.**
  5. A second incubation was done for 30 minutes at 37°C.
  6. After incubation, the washing step was repeated.
  7. Chromogen solution A followed by Chromogen solution B were added to each well, mixed and left for 15 minutes incubation at 37°C away from light.
  8. Stop solution was added to terminate the reaction generating a yellow end-product.
  9. Absorbance of color of each well was read immediately using a Microtiter Plate Reader setting its wavelength to 450 nm

**Calculation of results:**

The optical density (O.D) or absorbance of each calibrator was plotted versus the calibrator concentration to create a calibration curve using GraphPad Prism 9. The concentration of patient samples was then estimated from the calibration curve by interpolation**.**

**Supplementary tables:**

**Suppl. Table 1.** Laboratory findings of patients:

| **Laboratory data** | **Normal values** | **Mean ± SD** | **Median** | **Range** |
| --- | --- | --- | --- | --- |
| **LDH (mg/dL)** | 140-280 | 233.9 **±** 101.6 | 200.0 | (52.0-558.0) |
| **B2 micro-globulin** | 0.8-2.2 | 1.9 **±** 1.3 | 2.03 | (0.2-5.2) |
| **HDL (mg/dL)** | 35-70 | 49.9 **±** 23.1 | 48.0 | (15.0-147.0) |
| **Cholesterol (mg/d L)** | <200 | 208.3 **±** 41.7 | 207.0 | (128.0-336.0) |
| **TAG (mg/dL)** | <150 | 144.03 **±** 73.5 | 134.0 | (80.0-499.0) |
| **FBS** | 70-100 | 101.3 **±** 22.1 | 95.0 | (82.0-181.0) |
| **Hb (g/dl)** | 13.5-17.5 | 12.9 **±** 1.4 | 13.0 | (9.9-15.9) |
| **TLC (*10^3/cmm)** | 4.5-11.0 | 6.95 **±** 2.9 | 6.5 | (3.4-19.0) |
| **Platelets (*10^3/ul)** | 150-450 | 251.4 **±** 57.3 | 243.0 | (165.0-382.0) |
| **Lymphocytes (/cmm)** | 1000-4000 | 2365.9 **±** 781.3 | 2347.0 | (1083.0-4300.0) |
| **Monocytes (/cmm)** | 200-800 | 444.8 **±** 149.4 | 451.0 | (96.0-800.0) |
| **Eosinophils (/cmm)** | 0-500 | 117.03 **±** 74.02 | 107.0 | (0-325.0) |
| **Neutrophils (/cmm)** | 1500-8000 | 3613.3 **±** 1329.96 | 3550.0 | (1420.0-7090.0) |

SD: standard deviation, LDH: lactate dehydrogenase, HDL: high-density lipoprotein, TAG: triglyceride, FBS: fasting blood sugar, Hb: hemoglobin, TLC: total leukocytic count.

**Suppl. Table 2.** Diagnostic Accuracy Measures for IL-35 Tissue and Serum levels:

|  | **AUC** | **P value** | **95% CI** | | **Cut off** | **Sensitivity**  **%** | **Specificity**  **%** | **PPV**  **%** | **NPV**  **%** | **Accuracy**  **%** |
| --- | --- | --- | --- | --- | --- | --- | --- | --- | --- | --- |
|  |  |  | **Lower Bound** | **Upper Bound** |  |  |  |  |  |  |
| **IL-35 tissue level (ng/ml)** | 1.00 | <0.001 | 1.00 | 1.00 | 2.53 | 100 | 100 | 100 | 100 | 100 |
| **IL-35 serum level (ng/ml)** | 1.00 | <0.001 | 1.00 | 1.00 | 1.00 | 100 | 100 | 100 | 100 | 100 |

AUC: area under the curve, CI: confidence interval, PPV: positive predictive value, NPV: negative predictive value.

**Suppl. Table 3.** Comparisons of IL-35 tissue levels with different demographic and clinical data of MF patients:

| **Patients** | | **IL-35 tissue level (ng/ml)** | | | |
| --- | --- | --- | --- | --- | --- |
|  |  | **Mean ± SD** | **Median** | **Range** | **P value** |
| **Sex** | **Males** | (8.38 **±** 1.37) | 8.57 | (4.39-10.00) | **0.025** |
|  | **Females** | (9.39 **±** 0.98) | 9.40 | (7.38-10.69) |  |
| **Skin type** | **II** | (9.49 **±**.) | 9.49 | (9.49-9.49) | 0.369 |
|  | **III** | (9.44 **±** 0.81) | 9.49 | (8.27-10.62) |  |
|  | **IV** | (8.64 **±** 1.45) | 8.72 | (4.39-10.69) |  |
|  | **V** | (8.60**±.)** | 8.60 | (8.60-8.60) |  |
| **Recurrence** | **Yes** | (9.53 **±** 0.85) | 9.68 | (8.27-10.66) | **0.017** |
|  | **No** | (8.48 **±** 1.35) | 8.57 | (4.39-10.69) |  |
| **Type of lesion** | **Tumor** | (9.17 **±** 1.04) | 9.49 | (7.67-10.13) | 0.497 |
|  | **Plaque** | (8.64 **±** 1.18) | 8.54 | (6.72-10.66) |  |
|  | **Patch** | (9.04 **±** 1.35) | 9.21 | (4.39-10.69) |  |
| **Stage** | **IA** | (9.54 **±** 0.52) | 9.35 | (9.06-10.43) | 0.194 |
|  | **IB** | (9.08 **±** 0.92) | 8.59 | (7.98-10.69) |  |
|  | **IIA** | (8.34 **±** 1.83) | 8.68 | (4.39-10.66) |  |
|  | **IIB** | (9.55 **±** 0.71) | 9.75 | (8.57-10.13) |  |
|  | **IIIA** | (7.67**±**.) | 7.67 | (7.67-7.67) |  |

P<0.05 is statistically significant.

**Suppl. Table 4.** Comparisons of IL-35 serum levels with different demographic and clinical data of MF patients:

| **Patients** | | **IL-35 Serum level (ng/ml)** | | | |
| --- | --- | --- | --- | --- | --- |
|  |  | **Mean** ± **SD** | **Median** | **Range** | **P value** |
| **Sex** | **Males** | (7.97 ± 11.74) | 5.29 | (1.51-49.99) | 0.330 |
|  | **Females** | (6.91 ± 3.79) | 5.67 | (3.62-18.38) |  |
| **Skin type** | **II** | (8.27 ±.) | 8.27 | (8.27-8.27) | 0.556 |
|  | **III** | (6.53 ± 3.15) | 5.29 | (4.32-16.12) |  |
|  | **IV** | (7.93 ± 10.45) | 5.41 | (1.51-49.99) |  |
|  | **V** | (6.05 ±.) | 6.05 | (6.05-6.05) |  |
| **Recurrence** | **Yes** | (6.29 ± 2.96) | 5.67 | (3.40-16.12) | 0.883 |
|  | **No** | (8.27 ± 10.67) | 5.54 | (1.51-49.99) |  |
| **Type of lesion** | **Tumor** | (6.61 ± 2.01) | 6.86 | (4.32-8.81) | 0.526 |
|  | **Plaque** | (5.04 ± 1.71) | 5.29 | (1.51-6.86) |  |
|  | **Patch** | (8.54 ± 10.23) | 5.54 | (2.32-49.99) |  |
| **Stage** | **IA** | (7.13 ± 4.56) | 5.55 | (3.62-16.12) | 0.704 |
|  | **IB** | (8.16 ± 12.11) | 5.29 | (1.51-49.99) |  |
|  | **IIA** | (6.76 ± 4.37) | 6.32 | (2.32-18.38) |  |
|  | **IIB** | (6.55 ± 2.32) | 6.54 | (4.32-8.81) |  |
|  | **IIIA** | (6.86 ±.) | 6.86 | (6.86-6.86) |  |

P<0.05 is statistically significant.

**Suppl. Table 5.** Correlations between tissue IL-35 and studied variables:

| **Patients** | **IL-35 tissue level (ng/ml)** | |
| --- | --- | --- |
|  | **Correlation Coefficient** | **P value** |
| **IL-35 serum level (ng/ml)** | 0.046 | 0.794 |
| **Age** | -0.128 | 0.464 |
| **Duration (months)** | -0.050 | 0.775 |
| **BSA %** | -0.225 | 0.193 |
| **HDL** | 0.148 | 0.395 |
| **Cholesterol** | -0.119 | 0.495 |
| **TAG** | -0.143 | 0.411 |
| **FBS** | 0.001 | 0.996 |
| **LDH** | -0.045 | 0.799 |
| **B2 microglobulin** | -0.241 | 0.163 |
| **Hb (g/dl)** | 0.001 | 0.996 |
| **TLC (*10^3/cmm)** | -0.022 | 0.902 |
| **Platelets(*10^3/Ul)** | 0.178 | 0.308 |
| **Lymphocyes (/cmm)** | -0.157 | 0.367 |
| **Monocytes (/cmm)** | -0.412 | **0.014** |
| **Eosinophils(/cmm)** | 0.059 | 0.737 |
| **Neutrophils(/cmm)** | 0.113 | 0.518 |

P<0.05 is statistically significant, BSA: body surface area, HDL: high density lipoprotein, TAG: triglyceride, FBS: fasting blood sugar, LDH: lactate dehydrogenase, Hb: hemoglobin, TLC: total leucocytic count.

**Suppl. Table 6.** Correlations between serum IL-35 and studied variables:

| **Patients** | **IL-35 Serum level (ng/ml)** | |
| --- | --- | --- |
|  | **Correlation Coefficient** | **P value** |
| **IL-35 tissue level (ng/ml)** | 0.046 | 0.794 |
| **Age** | 0.155 | 0.373 |
| **Duration (months)** | 0.178 | 0.307 |
| **BSA %** | 0.052 | 0.767 |
| **HDL** | 0.132 | 0.450 |
| **Cholesterol** | -0.110- | 0.529 |
| **TAG** | -0.023- | 0.894 |
| **FBS** | -0.228- | 0.188 |
| **LDH** | -0.318- | 0.063 |
| **B2 microglobulin** | -0.036- | 0.839 |
| **Hb (g/dl)** | -0.146- | 0.403 |
| **TLC (*10^3/cmm)** | 0.114 | 0.515 |
| **Platelets(*10^3/Ul)** | 0.442 | **0.008** |
| **Lymphocytes(/cmm)** | 0.206 | 0.236 |
| **Monocytes(/cmm)** | -0.259- | 0.133 |
| **Eosinophils(/cmm)** | 0.229 | 0.186 |
| **Neutrophils(/cmm)** | -0.013- | 0.942 |

P<0.05 is statistically significant, BSA: body surface area, HDL: high density lipoprotein, TAG: triglyceride, FBS: fasting blood sugar, LDH: lactate dehydrogenase, Hb: hemoglobin, TLC: total leucocytic count.
